# Chemical Nature of Cod Liver Oil

**Published:** 1849-08

**Authors:** 


### CHEMISTRY.

*Chemical Nature of Cod Liver Oil.*—Dr. Jonathan Pereira has communicated to the Pharmaceutical Journal for February 1849, pp. 370–78, the important researches of De Jongh, accompanied by important remarks of his own. The following is an abridgment of the paper, which, our readers will observe (among other interesting facts,) points out that sulphuric acid is a test, by which the oil obtained from the livers of fish can be distinguished from the oil got from other parts of the animal.

The oils obtained from the livers of the tribe *Gadidæ* are very similar in their physical, and chemical, and probably also in their medicinal, properties. To all of them, the term *Oleum Jecoris Aselli, Oleum Jecoris Gadi*, or *Cod-liver Oil*, is indiscriminately applied; though it is commonly used to indicate the oil of the liver of the common cod (*gadus morrhua*, Cuv.) It would be better, therefore, to employ the term *Oleum Jecoris Morrhuæ*, when it is intended to designate the latter oil.

De Jongh, in his *Disquisitio Comparativa Chemico-Medica de Tribus Olei Jecoris Aselli Speciebus*, published at Leyden in 1843, states, that the Bergen (Norwegian) oil is principally obtained from three species: viz. the dorse (*gadus callarius*,) the coal-fish (*gadus carbonarius*,) and the pollack (*gadus pollachius*;) but chiefly from the first.

De Jongh made a very elaborate analysis of three kinds of Cod-liver Oil:—

1. *Pale Cod-liver Oil.*—Golden yellow; odor not disagreeable; not bitter, but leaving in the throat a somewhat acrid fishy taste; reacts feebly as an acid; sp. gr. 0·923 at 63°·5 Fahr. Cold alcohol dissolves from 2·5 to 2·7 per cent. of the oil; hot alcohol from 3·5 to 4·5 per cent.; in ether it is soluble in all proportions.

2. *Pale brown Cod-liver Oil.*—Color, that of Malaga wine; odor not disagreeable; bitterish, leaving a slightly acrid fishy taste in the throat; reacts feebly as an acid; sp. gr. 0·924 at 63°·5 Fahr. Cold alcohol dissolves from 2·8 to 3·2 per cent. of oil; hot alcohol, from 6·5 to 6·8 per cent. Ether dissolves it in all proportions.

3. *Dark brown Cod-liver Oil.*—Dark brown, in transmitted light greenish, in thin layers transparent; odour, disagreeable, empyreumatic; taste bitter and empyreumatic, leaving behind, in the fauces, an acrid sensation; reacts feebly as an acid; sp. gr. 0·929 at 63°·5 Fahr. Cold alcohol dissolves from 5·9 to 6·9 per cent, of it; hot alcohol, from 6·5 to 6·9 per cent. In ether it is soluble in all proportions.

De Jongh found the constituents of these oils to be *oleate* and *margarate of glycerine*, possessing the usual properties. But they also contained *butyric* and *acetic acids, the principal constituents of the bile* (bilifellinic acid, bilifulvin, and cholic acid,) *some peculiar principles* (among which was the substance called *gaduin*,) and not quite one per cent. of *salts*, containing iodine, chlorine, and traces of bromine. Moreover, he found that the oils contained free *phosphorus*.

The *pale* oil is richest in oleic acid and glycerine; the *brown* oil contains the largest amount of margaric, butyric, and acetic acids, and of the substances peculiar to Cod-liver oil; and the *pale brown* oil is richest in iodine and saline matters.

I. *Gaduin* (discovered by De Jongh) is thus obtained: Saponify Cod-liver Oil by means of caustic soda, and decompose the soap thus obtained by acetate of lead. The resulting lead-soap treat with ether, which takes up oleate of lead and gaduin, and leaves undissolved the margarate of lead. The ethereal solution is dark brown. If it be decomposed by sulphuric acid, brown oleic acid is set free. The brown colour of this acid is owing to the presence of gaduin. To separate the latter, add excess of caustic soda to the oleic acid, by which oleate of soda is formed. This is insoluble in the excess of caustic soda. It is to be dissolved in alcohol, and the alcoholic solution cooled below 32° Fahr., by which the oleate of soda separates, leaving, for the most part, the gaduin in solution. By sulphuric acid, the gaduin is precipitated from its solution.

Gaduin is first yellow, but on exposure to air, brown; it is odorless, tasteless, soluble in alcohol, but rendered insoluble by evaporating its solution to dryness. The alcoholic solution yields, on the addition of neutral acetate of lead, a copious precipitate, composed of $C_{35}$ $H_{22}$ O, P b O. If this lead salt be digested with carbonate of soda, it is decomposed, and a soda salt is obtained in solution, from which sulphuric acid precipitates a brown acid. Gaduin is insoluble in water, in nitric, and in hydrochloric acids. In sulphuric acid it dissolves, and acquires a blood-red color; but from this solution it is precipitated both by water and alkalies. It is soluble in alkalies. Diffused through water, and treated with chloride, it becomes decolorized. In burning, it yields an odor first of acetic acid, afterwards of cod-oil, and leaves behind a small quantity of ash. Berzelius thinks that gaduin is primitive bilifulvic acid; and that the reddish-brown substance, insoluble both in alcohol and water, which he (Berzelius) separated from bilifulvin by long and numerous operations, is only the insoluble modification of gaduin. Gaduin is contained in the three varieties of oil examined by De Jongh.

2. *Fatty acids,—margaric and oleic acids,*—do not appear to differ in their nature and composition from the same acids procured from other sources.

3. *Glycerine.*—This was obtained by saponifying Cod liver Oil by caustic soda.

4. *Bile Constituents.*—When Cod-liver Oil is shaken with water, an emulsion is obtained, from which the oil slowly separates. The aqueous liquid becomes clear by filtration. That which had been obtained by shaking the brown oil with water, was colored and empyreumatic; but the other kinds of oil did not color the water. The liquid invariably had a slightly acid reaction, and the oil which had been shaken with it was clearer, had a feebler odor, and reacted less powerfully as an acid. By boiling the oils with water, the same results were obtained. By evaporation, the aqueous fluids from all the three kinds of oil yielded a reddish-brown extract, which, softened by heat, was slightly soluble in water, more soluble in ether, and completely so in alcohol. Alkaline solutions dissolved it, and acids threw it down again in the form of a reddish-brown flocculent precipitate. The extracts had a peculiar odor, and a bitterish taste. The quantities obtained from the different kinds of oil were as follows:

| | With cold water. | With hot water. |
|---|---|---|
| Pale oil - - - | 0·607 per cent. - - - | 0.513 per cent. |
| Clear Brown oil | 0·890 — - - - | 0·849 — |
| Brown oil - - | 1·288 — - - - | 1·256 — |

When successively treated with ether, alcohol, and dilute spirit, all these extracts yielded the same results.

By ether, a reddish-brown, transparent, glutinous extract was obtained, which melted by heat, stained paper, and had the odor and taste of bile. After some time, small crystals made their appearance in it. It was slightly soluble in water, but readily so in ether and alcohol. A solution of carbonate of ammonia, added to its ethereal solution, caused separation into two layers. The upper turbid layer, by evaporation, yielded some drops of *olein*, some crystals of *margarin*, and a *brownish mass,* which was identical with that procured by evaporation of the lower layer. This brown mass had a bitter taste, was separated by water into a soluble and insoluble portion, and consisted of *fellinate* and *cholate of ammonia.*

The extract, which had been exhausted by ether, yielded to alcohol a blackish-brown, odorless, bitter, shining, hygroscopic mass, which dissolved with difficulty in water, and consisted of *biliverdin*, *bilifulvin*, and *bilifellinic acid.*

Dilute spirit removed from the residual extract a black shining substance, soluble in alkalies, concentrated sulphuric acid, and hot acetic acid, but insoluble in nitric and hydrochloric acids. From its alcoholic solution, baryta-water and acetate of lead precipitated it of a brown color. It left no residue by burning.

The residue of the aqueous extract, left after the action of the three above mentioned solvents, contained an *organic substance* (whose nature has not been determined) and *inorganic salts*, in which chlorine, phosphoric and sulphuric acids, lime, magnesia, and soda were found, but no potash or iodine.

5. *Iodine, bromine, and chlorine.*—Their therapeutical agency in the oil must, if any, be exceedingly small the proportions in which they exist are inconstant, though very trifling. Beneficial effects have been produced by the use of the oil, which neither iodine nor bromine are capable of producing. De Jongh says, that iodine is present in genuine Cod-liver Oil, but that the only certain mode of detecting it is to saponify the oil, and carbonize the resulting soap. He confirms Stein's remark, that neither by immediately carbonizing the oil, nor by saponifying it, and then decomposing the soap by acids, can the iodine be detected. It follows, therefore, that iodine exists in the oil neither in the free state nor in that of metallic iodine, but probably in organic combination—perhaps, as an iodic fatty acid. De Jongh determined the proportion of iodine by forming iodide of palladium: every 100 parts of anhydrous iodide of palladium was considered equivalent to 70.34 parts of free iodine.

The largest amount of iodine found in genuine oil is less than 0.05 per cent. If the amount obtained be larger than this, fraud may be suspected. Dr. Martini says, that dishonest druggists have introduced iodine into the oil, to augment its commercial value; and it is stated that artificial Cod-liver Oil has been made by combining iodine with common train oils.

De Jongh detected *bromine* and *chlorine,* in minute quantities, in the oil.

*Phosphoric and sulphuric acids, and Phosphorus,* were found by De Jongh.

7. *Acetic and Butyric acids.*—De Jongh separated these volatile acids from Cod-liver Oil by adding sulphuric acid to the soda-soap, and distilling the liquor thus obtained.

The Genuineness and Purity are known partly by physical, and partly by chemical tests.

The Physical Characters are color, odor, and flavor. The finest oil is most devoid of color, odor, and flavor. The oil in the cells of the fresh liver is nearly colorless, and the brownish color possessed by the ordinary Cod-oil used by curriers is due to coloring matters derived from the decomposing hepatic tissues and fluids, or from the action of air on the oil. Chemical analysis lends no support to the opinion, at one time entertained, that the brown oil was superior as a therapeutical agent, to the pale oil. If patients could conquer their aversion to the brown oil, its free use, like that of other rancid and empyreumatic fats, would often disturb the digestive functions, and be attended with injurious effects.

Of the Chemical Characters which have been used to determine the genuineness of Cod-liver Oil, some have reference to the iodine, others to the gaduin, or to the bile constituents. Iodine, or iodide of potassium, added to train oil, to imitate Cod-liver Oil, may be readily detected by adding a solution of starch and a few drops of sulphuric acid, by which the blue iodide of starch is produced; or the suspected oil may be shaken with alcohol, which abstracts the iodine. But though we may thus readily prove that the suspected oil contains no artificially added iodine, the iodine which is naturally contained in, and more intimately combined with the oil, may be frequently recognized by another process. Marchand gives the following directions for detecting it: Saponify the oil wi'h soda, carbonize the soap thus obtained, digest the coal in distilled water, add a drop of starch paste, and subject the mixture to the action of a voltaic battery, the positive pole being placed in contact with the starch paste, the negative pole with the solution. If iodine be present the starch becomes blue. Marchand states that by this test, the iodine can be detected in the urine of a patient soon after he has taken the oil. This, however, is certainly not always correct; for I submitted the urine of a young gentleman, who, for several weeks, had taken with great benefit a table spoonful of Cod-liver Oil thrice daily, to the action of a galvanic battery of fifty pairs of plates for several hours, without obtaining the slightest evidence of the presence of iodine.

Sulphuric acid has been employed as a test for Cod-liver Oil. If a drop of concentrated sulphuric acid be added to fresh Cod-liver Oil, the latter assumes a fine violet color, which soon passes into yellowish or brownish-red. Some samples produce at once the red color, without the preliminary violet tint. It has been erroneously supposed by some persons, that this violet color was due to the evolution of iodine, by the action of the acid on an alkaline iodine contained in the oil. If that were the case, the presence of a little starch-paste would be sufficient to convert the violet into an intense blue color; which is not the case. The coloration in fact depends on the action of the sulphuric acid on some one or more organic constituents of the oil, and the following facts lead me to infer that it is in part due to the presence in the oil of one of the constituents of the bile. In 1844, Pettenkofer pointed out a new test for bile. If to a liquid supposed to contain bile, about two-thirds of its volume of oil of vitriol be added, the liquid kept cool, a few drops of a solution of cane-sugar (four or five parts of water to one of sugar) be added, and the mixture shaken up, a violet red color is produced, provided bile be present. This test succeeds very well, if we dissolve a little extract of ox-bile in water, and test the solution with sugar and oil of vitriol. The color developed agrees with that produced by the oil of vitriol to Cod-liver Oil, which De Jongh has shown, contains the essential constituents of the bile. Pettenkofer remarks, that the presence of a very great excess of chlorides will change the violet-red color into a brownish-red. This fact is deserving of notice, because it may aid in accounting for the fact that some specimens of Cod-liver Oil strike a brownish-red, not a violet-red color, with oil of vitriol.

Strecker confirms Platner's observation that both cholic and paracholic acids produce the same color with sugar and oil of vitriol, as bile does; so that Pettenkofer's test doubtless acts on one or both of these acids. Now De Jongh has shown that cholic acid is contained in Cod-liver Oil, and we have, therefore, good reason for believing that it is in part by the action of oil of vitriol on this acid, that the violet-red color is produced. For the development of this color in bile it is necessary to use, besides oil of vitriol, a third agent (sugar.) For cane-sugar we may substitute grape-sugar, starch, or any substance which can by the action of oil of vitriol be converted into grape-sugar. No such substance has hitherto been detected in Cod-liver Oil, and, therefore, it may be said the necessary ingredient to produce this characteristic re-action of oil of vitriol on cholic acid is wanting. Strecker has recently supplied the wanting link. In his valuable paper on ox-bile, he observes that acetic acid may be substituted for sugar. To the liquid supposed to contain bile, add a few drops of acetic acid, and then concentrated sulphuric acid, when a magnificent purple-red color is developed. If the quantity of bile be small, it may be necessary to use heat. Now, as Cod-liver Oil contains acetic acid, we have the requisite agent to enable the oil of vitriol to act on the cholic acid, and the development of the purple or violet-red color is then readily accounted for. I have already noticed the red color produced by the action of oil of vitriol on gaduin (supposed by Berzelius to be derived from the bile.) Here, then, is another source for the red color caused by the action of sulphuric acid on Cod-liver Oil.

Sulphuric acid, then, is a test for liver oils. It does not distinguish one liver oil from another: neither does it distinguish good Cod-liver Oil from bad, for it produces its characteristic re-action both with common brown Cod-oil and with the finest and palest qualities. But it serves to distinguish oil procured from the liver, from oil obtained from other parts of the animal.—*Lond. Jour. of Med.*

